# Supplementary material for: Curli-independent defense against Bdellovibrio bacteriovorus in E. coli
Source: Microbiol Spectr. 2025 Sep 29;13(11):e00342-25. doi: 10.1128/spectrum.00342-25 (PMC12584612; doi:10.1128/spectrum.00342-25)
Supplement: Figures S1 to S4 — Fig. S1: Group 2 transposon mutants B. bacteriovorus susceptibility. Fig. S2: Marked deletions in lacZ and ACS228_15805 do not impact ECOR29 defense. Fig. S3. Deletions in Group 1 genes proC, tolC, and glpX did not impact ECOR29. Fig. S4: Unedited transmission electron microscopy images. [file spectrum.00342-25-s0001.pdf]

SUPPLEMENTAL FIGURES

Curli-independent defense against *Bdellovibrio bacteriovorus* in *E. coli*

Ryan Sayegh[a,b], Hannah E. Ledvina[a], Aaron T. Whiteley[a#]

[a] Department of Biochemistry, University of Colorado Boulder, Boulder, CO, USA
[b] Department of Molecular, Cellular and Developmental Biology, University of Colorado Boulder, Boulder, CO, USA

Running title: Curli-independent *Bdellovibrio* defense

#Address correspondence to Aaron Whiteley, aaron.whiteley@colorado.edu

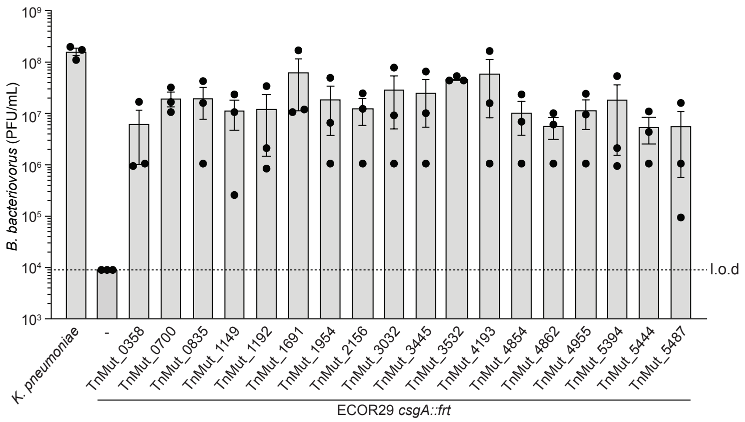

Figure S1: Group 2 transposon mutants *B. bacteriovorus* susceptibility. Efficiency of plating, calculated as PFU/mL, of *B. bacteriovorus* HD100 when infecting indicated bacterial strain. Limit of detection (l.o.d.) indicated by dotted line. Data are the mean ± standard error of the mean for n=3 biological replicates.

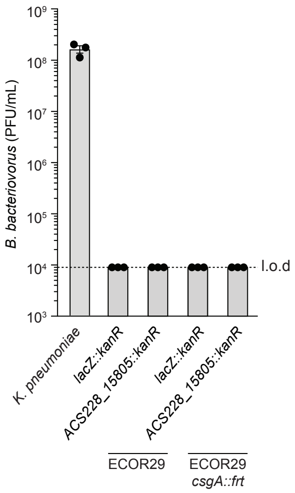

Figure S2. Marked deletions in *lacZ* and *ACS228_15805* do not impact ECOR29 defense. Efficiency of plating, calculated as PFU/mL, of *B. bacteriovorus* HD100 when infecting indicated bacterial strain. Data graphed as in **Figure S1.**

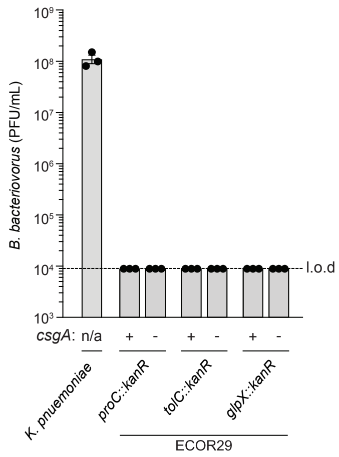

Figure S3. Deletions in Group 1 genes *proC*, *tolC*, and *glpX* did not impact ECOR29 defense. Efficiency of plating, calculated as PFU/mL, of *B. bacteriovorus* HD100 when infecting indicated bacterial strain. Data graphed as in **Figure S1.**

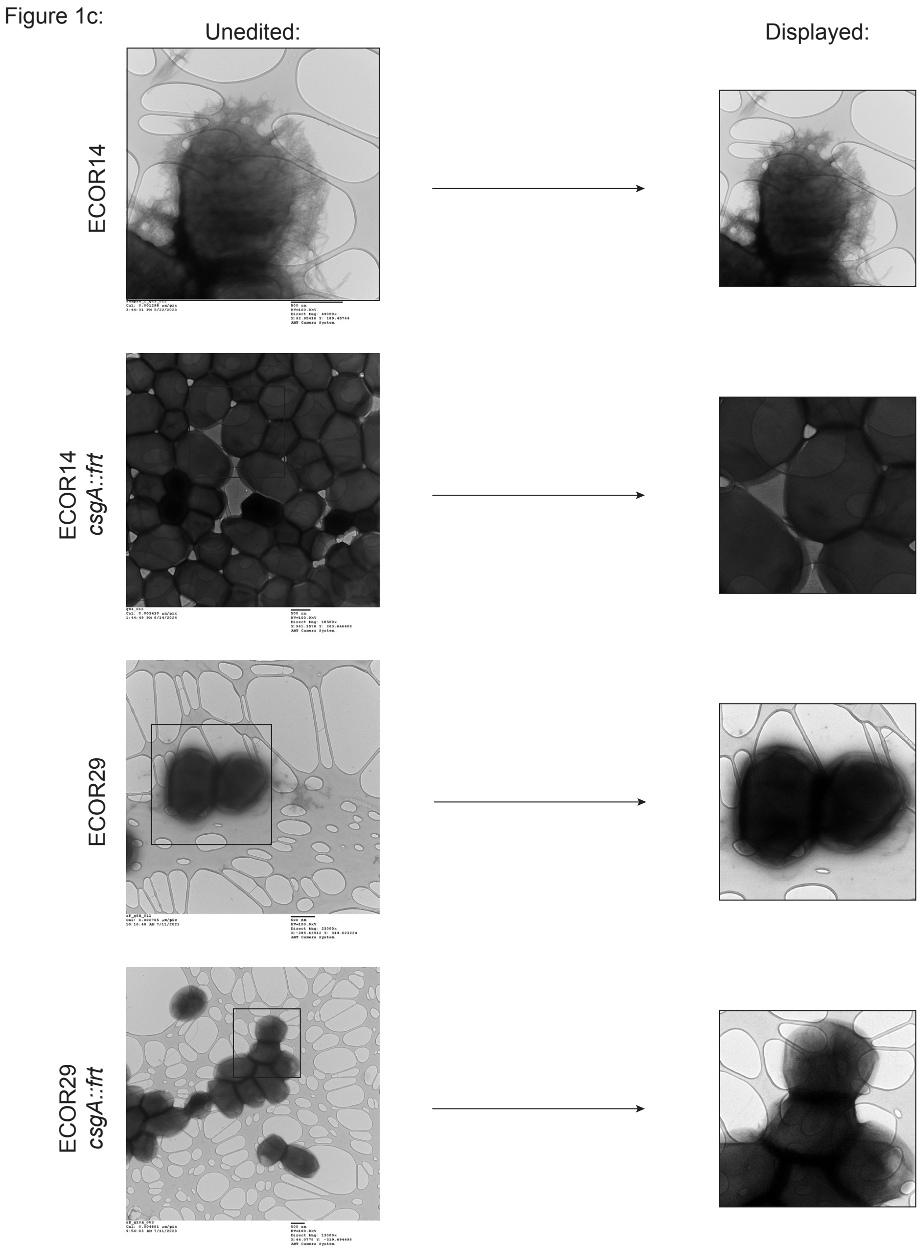

Figure S4: Unedited transmission electron microscopy images. Unedited transmission electron microscopy images along with the cropping applied to each image

Figure 4a:

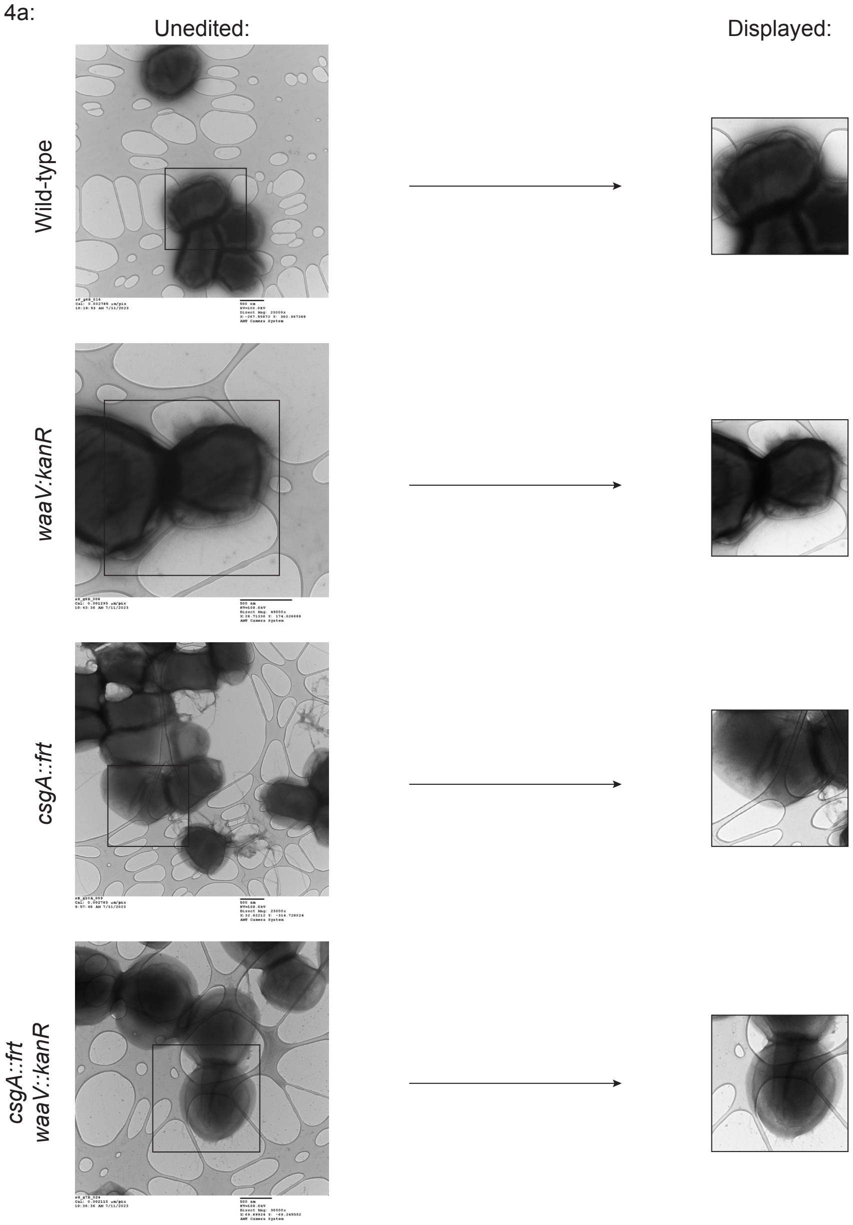

Figure S4: Unedited transmission electron microscopy images. Unedited transmission electron microscopy images along with the cropping applied to each image
